# Collateral Benefit of COVID-19 Control Measures on Influenza Activity, Taiwan

**DOI:** 10.3201/eid2608.201192

**Published:** 2020-08

**Authors:** Shu-Chen Kuo, Shu-Man Shih, Li-Hsin Chien, Chao A. Hsiung

**Affiliations:** National Health Research Institutes, Zhunan, Taiwan

**Keywords:** coronavirus disease, SARS-CoV-2, severe acute respiratory syndrome coronavirus 2, viruses, respiratory infections, zoonoses, COVID-19, Taiwan, influenza, infection control measures

## Abstract

Taiwan has strictly followed infection control measures to prevent spread of coronavirus disease. Meanwhile, nationwide surveillance data revealed drastic decreases in influenza diagnoses in outpatient departments, positivity rates of clinical specimens, and confirmed severe cases during the first 12 weeks of 2020 compared with the same period of 2019.

After the 2003 severe acute respiratory syndrome coronavirus epidemic, the government and public of Taiwan have been vigilant about the threat of emerging infectious diseases. The government of Taiwan took swift action to prevent coronavirus disease (COVID-19) importation and outbreaks (*1*). The public has adhered well to control measures that included avoiding gatherings, maintaining social distance, mask wearing, hand and respiratory hygiene, temperature monitoring, and quarantine of high-risk and sick persons (Figure, panel A). Although the success of these measures for limiting COVID-19 transmission remains to be determined, nationwide surveillance has shown the rapid decline of influenza activity during the first 12 weeks of 2020 (through March 21) in Taiwan.

**Figure:**
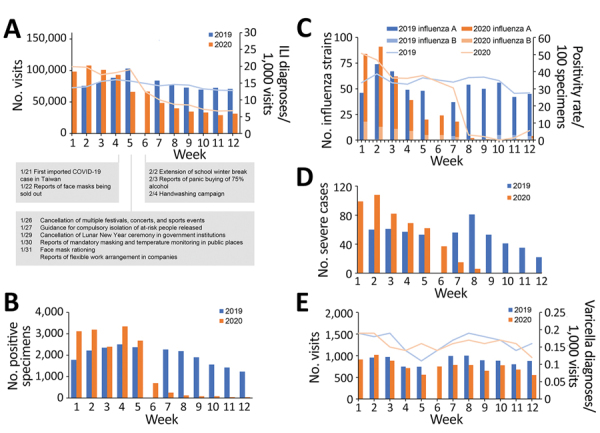
Influenza and varicella activity in Taiwan during the first 12 weeks of 2020 compared with the corresponding time period in 2019. A) Number of outpatient department visits in which the diagnosis of influenza-like illness (ILI) was made (bars) and the rate of ILI diagnoses per 1,000 visits (lines). Notable dates during the coronavirus disease pandemic are marked along the baseline. B) Number of specimens positive for influenza. C) Number of strains of influenza identified in commissioned laboratories (bars) and the number of positive specimens/total specimens positivity rate (lines). D) Number of laboratory-confirmed influenza cases with severe complications. E) Number of outpatient department visits in which the diagnosis of varicella infection was made (bars) and the rate of varicella diagnoses per 1,000 visits (lines). The 9-day Lunar New Year holiday in week 6 of 2019, when most healthcare service was unavailable, resulted in extreme data, which we excluded from the analysis.

The Taiwan National Infectious Disease Statistics System (*2*), maintained by the Taiwan Centers for Disease Control, is an open data portal that provides nationwide surveillance data on infectious diseases (https://nidss.cdc.gov.tw). For this study, we analyzed data from outpatient department visits for selected syndromes, clinical specimen testing, isolated respiratory pathogens, and confirmed severe cases (Appendix). The institutional review board of the National Health Research Institutes approved this study (EC1051207-R4).

We compared changes across the first 12 weeks of 2020 with data from the same period of 2019 using simple linear regression. (The 9-day Lunar New Year holiday in week 6 of 2019, when most healthcare service was unavailable, resulted in extreme data; therefore, we excluded these data from analysis.) We saw fewer outpatient department visits for influenza-like illness (ILI) and ILI diagnoses per 1,000 visits during weeks 8–12 of 2020 compared with 2019 (Figure, panel A). The changes (slopes of the regression lines) of ILI visits (−8,061 vs. −590 per week; p<0.05) differed between 2020 and 2019, as did the changes in ILI diagnoses per 1,000 visits (−1.5 vs. −0.2 per week; p<0.05). The slopes of the regression lines for positive samples (−360 vs. −77 per week; p<0.05) also differed between 2020 and 2019 (Figure, panel B). Both the number of influenza strains isolated from clinical specimens in commissioned laboratories and the positivity rate dropped drastically in 2020; the trends were different from 2019 (p<0.05 for both) (Figure, panel C). The number of cases of confirmed influenza with severe complications decreased from 99 to 1 in 2020, compared with a decrease from 44 to 22 in 2019 (p<0.05) (Figure, panel D). In contrast, the number of outpatient department visits for varicella and the number of varicella diagnoses per 1,000 visits remained similar in 2020 and 2019 (p = 0.660 for outpatient department visits and p = 0.157 for varicella diagnosis) (Figure, panel E). 

The functional healthcare and surveillance systems in Taiwan, the government’s efforts to identify causes of ILI during the COVID-19 pandemic, and sufficient laboratory capacity ensure appropriate influenza testing and reporting of results. Healthcare avoidance during COVID-19 pandemic may be an important confounder for the results we reported. However, because of awareness of the similarities in symptoms between COVID-19 and influenza and the low number of COVID-19 patients in Taiwan (<200 cases as of March 21, 2020), patients with ILI would not avoid seeking medical help for a diagnosis. Healthcare avoidance also did not explain the lower number of severe influenza cases observed in 2020 (Figure, panel D). Therefore, we believe that the decreasing influenza activity in Taiwan in 2020 is the result of strict control measures that were established in response to COVID-19.

Appendix Additional information about collateral benefits of COVID-19 control measures on influenza activity in Taiwan.
