# Supplementary material for: Collateral Benefit of COVID-19 Control Measures on Influenza Activity, Taiwan
Source: Emerg Infect Dis. 2020 Aug;26(8):1928–30. doi: 10.3201/eid2608.201192 (PMC7392415; doi:10.3201/eid2608.201192)
Supplement: Appendix — Additional information about collateral benefits of COVID-19 control measures on influenza activity in Taiwan. [file 20-1192-Techapp-s1.pdf]

Article DOI: https://doi.org/10.3201/eid2608.201192

Collateral Benefit of COVID-19 Control Measures on Influenza Activity, Taiwan

Appendix

Methods

We used 4 datasets from Taiwan National Infectious Disease Statistics System (TNIDSS) in this study. These datasets provided (1) the number of outpatient department (OPD) visits for selected syndromes and the number of all OPD visits; (2) the number of clinical specimens that tested positive for influenza; (3) the number of specimens sent to commission laboratories and the species of respiratory pathogens isolated; and (4) the number of severe complicated cases with confirmed influenza.

Aggregated data regarding OPD visits (the first dataset) are from the National Health Insurance of Taiwan. International Classification of Diseases (ICD-9-CM/ICD-10) codes were used to identify influenza-like illness and varicella. Data on specimens positive for influenza (the second dataset) were obtained from >50 clinical laboratories. Some of the specimens were sent to 8 commissioned laboratories for virus culture and species identification; experimental results (the third dataset) were then submitted to TNIDSS. Reporting of cases of suspected influenza with severe complications is mandatory; they were reported to Taiwan Centers for Disease Control and specimens were sent to the CDC laboratory for confirmation (the fourth dataset).
